# Increasing patient comfort in palliative radiotherapy with a newly developed mattress: a nonrandomized clinical trial

**DOI:** 10.1016/j.ctro.2025.101017

**Published:** 2025-07-16

**Authors:** Nienke Hoffmans-Holtzer, Britt Kunnen, Olijn Tims, Ilse de Pree, Cleo Slagter, Manouk Olofsen-van Acht, Mischa Hoogeman, Steven Petit

**Affiliations:** Erasmus MC Cancer Institute, University Medical Center Rotterdam, Department of Radiotherapy, P.O. Box 2040, 3000 CA Rotterdam, Netherlands (the)

**Keywords:** RTComfort, Radiotherapy, Radiation oncology, Palliative radiotherapy, Comfort, Mattress

## Abstract

**Background:**

In palliative radiotherapy, many patients experience discomfort and pain during treatment, particularly while lying on flat, hard treatment couches that are considered essential for accurate treatment delivery. Thin foam matts, often used for palliative treatments, can somewhat reduce discomfort, but they are frequently insufficient.

**Aim:**

In this study a mattress was developed and investigated, with the aim to reduce pain during radiotherapy treatments without affecting treatment quality.

**Methods:**

A nonrandomized clinical trial compared the newly designed RTComfort mattress with a standard thin foam matt. The primary endpoint was mattress preference, while secondary endpoints included experienced pain, positioning stability, and dosimetric effects. Included patients tested both the mat and mattress for one minute each in the treatment position before planning-CT acquisition, scoring their preference and pain on a Numerical Rating Scale (NRS, 0–10). Subsequently, patients were scanned and treated on their preferred option. Positioning stability during treatment was evaluated using optical surface scanning and dosimetric effects were evaluated through dose calculations with the treatment planning system.

**Results:**

Out of 45 patients, 44 (98 %) preferred the RTComfort mattress, with 35 patients showing a strong preference (p < 0.0001). The median pain score on the RTComfort mattress decreased from 4.8 (IQR: 2.0–6.1) to 2.0 (IQR: 0.4 to 4.0 NRS) (p < 0.0001). Both options showed negligible patient sagging, with marginally less on the RTComfort mattress (p < 0.0001). No relevant dosimetric effects were observed.

**Conclusions:**

This prospective clinical trial highlights the need for more comfortable radiotherapy treatments. Compared to the standard thin foam matt, 98% of patients preferred the RTComfort mattress. The RTComfort mattress provided clinically significant reduction in pain associated with lying on flat, hard treatment couches. The mattress is safe for clinical use and shows potential beyond palliative radiotherapy settings.

## Highlights

• Flat and hard treatment tables can cause discomfort and pain during radiotherapy.
• The new RTComfort mattress adds comfort without affecting positioning and dosimetry.
• This clinical study showed that a great majority preferred the RTComfort mattress.
• Median pain scores were significantly reduced, both statistically and clinically.
• Findings support further deployment, also for curative (online adaptive) treatments.

## Introduction

The World Health Organization estimated the worldwide cancer incidence to 20 million new cases in 2022, with a predicted increase of 77 % in 2050 [1]. Over 50 % of new cancer cases require radiotherapy as their definitive or adjuvant therapy [2]. Approximately half of all radiotherapy treatments (i.e.: roughly 5 million per year) are delivered in palliative setting, to offer acute benefit by alleviating symptoms related to advanced cancer [[3], [4], [5]].

In radiotherapy, patients are typically positioned on flat and hard treatment couches to ensure a reproducible patient position during the different treatment fractions. Each treatment fraction lasts 10 to 15 min, which can cause discomfort and (additional) pain, especially in patients receiving palliative radiotherapy who are often frail and in pain already [[6], [7]]. Discomfort, along with feelings of anxiety, restlessness, and pain can compromise the radiotherapy treatment quality [8]. To achieve a bit less discomfort, patients treated with palliative radiotherapy are therefore usually positioned and treated on a thin foam matt. Historically, these matts were designed to be thin, approximately 2 cm, for two reasons. First, to prevent undesirable attenuation of the treatment beams causing an increase in radiation dose deposited to the skin [9]. Second, to minimize day-to-day variation in patient positioning, negatively affecting the precision of the radiotherapy treatment. Unfortunately, existing matts are too thin to effectively counter discomfort and pain in patients treated for already painful bone metastasis [[10], [11], [12]]. A recent article of Goldworthy et al. (2023) [12] proposed the development of a soft mattress to alleviate discomfort as an important strategy to potentially improve radiotherapy treatment accuracy and efficacy and addressed that this new soft mattress should be thoroughly evaluated.

For the development of such a soft mattress for patient positioning, patient comfort and treatment quality should be balanced. In the last decade, volumetric intensity modulated radiotherapy (VMAT) has become more mainstream in modern palliative radiotherapy [[13], [14], [15], [16]]. As VMAT delivers radiation from a large range of angles and varying intensities, delivered dose is less dependent on the presence/absence of a mattress. Moreover, to further limit the maximum skin dose or increase target dose coverage, VMAT plans can be optimized for the mattress presence. Potentially, this allows for use of a thicker mattress to enhance patient comfort without compromising the treatment quality. To leverage this opportunity, a brainstorm session a was conducted between radiotherapy professionals and mattress manufacturers. The outcome of this session was a set of key design criteria leading to an ideal mattress for palliative radiotherapy. It should (i) be compatible with existing radiotherapy positioning devices; (ii) easy to clean; (iii) not negatively affect patient positioning stability; (iv) have negligible impact on skin dose and (v) negligible attenuation of the radiation beam. Naturally, (vi) it should reduce experienced pain while lying on the treatment couch. Currently, to the best of our knowledge, there are no such mattresses available [12].

Therefore, the aim of this study was to develop and evaluate a new radiotherapy mattress that meets the six requirements described above. Iteratively, a series of prototypes was fabricated and tested using phantoms. Next, the best prototype was released for clinical use and tested in a prospective observational study with 45 patients treated with palliative radiotherapy for bone metastasis.

## Material and methods

### Mattress development

To develop a new mattress, a series of prototypes was fabricated, which was compatible with the treatment table and positioning devices used in our institute (i.e.: MacroMedics®) (requirement (i)). The prototypes did not contain metal parts, and to ensure optimal endurance and hygiene, a mattress ticking fabric was selected which is commonly used at intensive care settings (requirement (ii). Requirements regarding treatment quality (iii) to (v) were evaluated for a subset of prototypes in phantom based CT-measurements (Speidel Round Plastic Fermenter, 20L), and optical surface scanning measurements of volunteers (AlignRT®, version 6.3, VisionRT Ltd, London, UK). See also Figs. S1 and S2 in the Supplementary materials. From this subset, the mattress of choice was released for clinical practice and was named the RTComfort mattress (patent pending). This RTComfort mattress consists of two distinct layers of foam and a water resistant bi-stretch tike. The mattress has an uncompressed thickness of 6 cm.

Of note, since the mattress has no active interaction with patients (i.e.: the patients are not forced in any position) and any potential impact of the RTComfort mattress on the treatment delivery is considered negligible, the new mattress is not classified as a medical device. Consequently, there were no specific guidelines governing the development and application of this type of mattress.

### Clinical study

To determine whether the RTComfort mattress indeed could increase patient comfort with respect to the standard matt (requirement (vi)) a prospective clinical study was performed. This study was approved by the Institutional Review Board (number: MEC-2023-0295), and registered at ClinicalTrials.gov (number: NCT06903507). Only Dutch speaking patients with age ≥16 were included who were treated with palliative radiotherapy for bone metastasis in thoracic, abdominal and/or pelvis region, at our one-stop shop outpatient clinic between August and December 2023. Fractionations schemes included 1 × 8 Gy, 2 × 8 Gy, 4 × 5 Gy, and 10 × 3 Gy. To offer optimal comfort, all patients were treated in supine position [17]. The primary objective was to determine if patients preferred the RTComfort mattress over the standard matt. As secondary objectives, this study assessed the level of mattress preference (slight or strong) and the experienced pain intensity on both mattresses. Also, the mattress sagging was evaluated by measuring the patient’s positioning stability during treatment, using optical surface scanning (requirement (iii)). Finally, treatment plans were recalculated with and without mattress to confirm the expected negligible effect on skin dose and attenuation (requirements (iv) and (v)).

#### Study procedures

During the intake with the radiation oncologist, patients were asked informed consent to participate in the study. Prior to CT scan acquisition, the included patients tested the regular matt and the RTComfort mattress by lying on each for one minute in radiotherapy position, on the flat CT scanner couch. During the testing, patients scored pain intensity according to the Numerical Rating Scale (NRS, 0–10) [18], expressed preference for the RTComfort mattress or the standard matt, and whether the preference was slight or strong. To mitigate any potential bias, the first 22 patients tried out the standard matt first and afterwards the RTComfort mattress, while the sequence was reversed for patients 23 to 45. Next, the treatment preparation and delivery were performed according to standard clinical practice, with the patient positioned on the matt or mattress of choice, as described in the following section.

#### Radiotherapy treatment preparation and delivery

CT scanning, delineation, treatment planning and treatment delivery were all performed according to standard clinical practice. In short, the patients were scanned on the CT scanner (SOMATOM Confidence, Siemens, Erlangen, Germany), positioned on the matt or mattress of choice. During treatment planning, the RTComfort mattress was not taken into account, as phantom experiments showed it had a negligible impact on the treatment plan. On the treatment couch, prior to treatment, patients were again positioned onto the mattress of choice. During positioning and treatment, the patient external was monitored using the AlignRT® optical surface guidance system with a focus around the region where the tumor was located (e.g. around the hips for pelvic patients, thymus for thoracic patients) with a resolution of approximately 21 Hz. As optical surface guidance was used, no difference was expected between setup time and handling on the RTComfort mattress compared to the standard foam matt.

#### Mattress preference: sample size calculation

To determine whether most patients preferred the RTComfort mattress (primary objective), a sample size of 36 patients was required based on a one-sample proportions z-test with 70 % estimated preference for RTComfort mattress, ±15 % error margin and a 95 % confidence level. Since it was foreseen that for some patients the optical surface monitoring system may not be able to accurately detect the body outline of the patients, for instance due to too much body hair, accrual continued until either 36 patients were included that could be evaluated for all objectives up to a maximum number of 45 in total.

### Data analysis

#### Patient positioning stability

The positioning stability of the patients included in the clinical study was determined using AlignRT® (version 6.3, VisionRT Ltd, London, UK) and expressed as the vertical position of the patients’ surface over time; all AlignRT® measurements were started directly after CBCT positioning verification and stopped directly after dose delivery. The positioning stability on the mattress was considered sufficient if two requirements were met; (1) the sagging (i.e.: the change in vertical position over time) during the first 5 min of dose delivery should be less than 1 mm averaged over all patients, (2) the variation in vertical position over time should not be larger compared to patients positioned during treatment on the standard matt. As the number of included patients preferring the RTComfort mattress appeared much higher than the number of included patients preferring the standard matt, little data was available regarding positioning stability during treatment on the standard matt. Therefore, the positioning stability of the patients on the RTComfort mattress was compared to a historic reference cohort of similar patients positioned on the standard matt, acquired between October 2019 and October 2021 with AlignRT® version (version 5.1.2).

During rotational VMAT dose delivery, the treatment machine could temporally obscure the ROI from the AlignRT® cameras. Data points with less than 50 % of the ROI captured due to camera obscuring could cause unreliable outcomes and were therefore excluded. To enable comparison between both groups and to mitigate breathing motion the data was binned with a 6-s bin-size. The median of the bins was used for further analysis. The statistical analysis is described in the statistical analysis section.

#### Dosimetric evaluation

As negligible dosimetric effect was expected, the original treatment plan was calculated without taking the RTComfort mattress taken into account. To investigate the expected negligible effect of the mattress attenuation on 95 % dose coverage of the planning target volume (PTV) and skin dose, the treatment plans were recalculated and reoptimized while accounting for the RTComfort mattress using the Monte Carlo algorithm of Monaco 6.00.01 (grid spacing of 0.3x0.3x0.3 cm^3^, statistical uncertainty of 1 % per calculation). The skin contour was defined as the outer 5-mm of the patient body contour for all CT-slices that included a PTV contour. The PTV itself was excluded from the skin structure.

### Statistical analysis

To evaluate the mattress preference (standard matt versus RTComfort mattress), a one-sample proportion Z-test was applied with a sigma of 0.5. To evaluate any potential bias caused by whether the patient first tried out on the standard matt or the RTComfort mattress, these two groups were split in “RTComfort First” and “Standard First”, respectively. Next, for each mattress separately, the pain scores between the “RTComfort First” and “Standard First” were tested for statistical differences using a two-sided Mann Whitney *U* test. Next, a Wilcoxon signed-rank was performed to compare pain scores for all patients for the standard matt versus the RTComfort mattress. Both tests were performed using SciPy (version 1.10.1) with Python (version 3.8.10).

The positioning stability of patients on the RTComfort mattress during treatment was compared to a historical cohort of patients positioned on the standard matt in terms of average vertical position per patient group. For that purpose, linear regression analysis was applied as implemented in statsmodels (version 0.13.0) in Python (version 3.8.10). The average vertical position of both patient groups was combined in a single dataset, with the dependent variable being the average vertical patient position as a function of time. The independent variables were the *time*, the *type of mattress* and the interaction term time*type of mattress. P values < 0.05 were considered statistically significant.

## Results

### Mattress development

In total six prototypes were tested by repetitive CT-measurements of the pre-heated water phantom. Three prototypes could fulfil requirements (i) to (v), including dosimetric equivalence, of which the one that felt most comfortable was selected for volunteer measurements. After clinical approval, this RTComfort mattress was used in the study to test requirement (vi). Results regarding the mattress development are available in the Supplementary Materials (Figs. S3 and S4, Table S1).

### Clinical study

#### Mattress preference and pain intensity scores

**Fig. 1:**
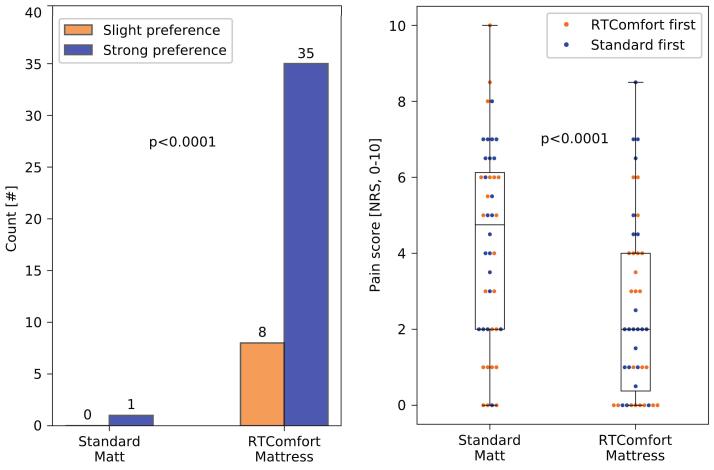
Questionnaire results about mattress preference and experienced pain scores. The left panel shows the mattress preference for 44 patients (slight preference in orange, strong preference in blue). For one patient the level of preference was unknown, however, this patient did prefer the RTComfort mattress over the standard matt. The right panel shows the pain intensity scores for individual patients (dots) on the standard matt and on the RTComfort mattress. The orange dots are scored by the group of patients who were positioned on the RTComfort mattress first (n = 22), the blue dots by patients who were positioned on the standard matt first (n = 22). The boxes indicate the inter-quartile range (IQR) between Q1 and Q3, the whiskers extend from the box to the farthest data point lying within 1.5 times the IQR from the box of the entire cohort. (For interpretation of the references to colour in this figure legend, the reader is referred to the web version of this article.)

From the 45 patients in the clinical study, 44 filled-out questionnaires could be collected. In total, 44 patients preferred the RTComfort mattress over the standard matt, with 35 patients expressing strong preference (p < 0.0001, see Fig. 1A). Fig. 1B shows the pain scored by the patients while trying out both mattresses right before planning CT scan acquisition. Mann Whitney U tests showed no significant differences between groups “Standard first” and “RTComfort first” on the standard matt (p = 0.18), nor on the RTComfort mattress (p = 0.35), indicating no bias between the groups. Wilcoxon signed-rank tests showed that patients experienced statistically significantly less pain on the RTComfort mattress compared to the standard matt; the median pain score reduced by 2.8 points (p < 0.0001) from 4.8 (IQR: 2.0–6.1) to 2.0 (IQR: 0.4 to 4.0 NRS) respectively. Note that this reduction in pain score is also considered clinically significant [18].

#### Patient positioning stability

**Fig. 2:**
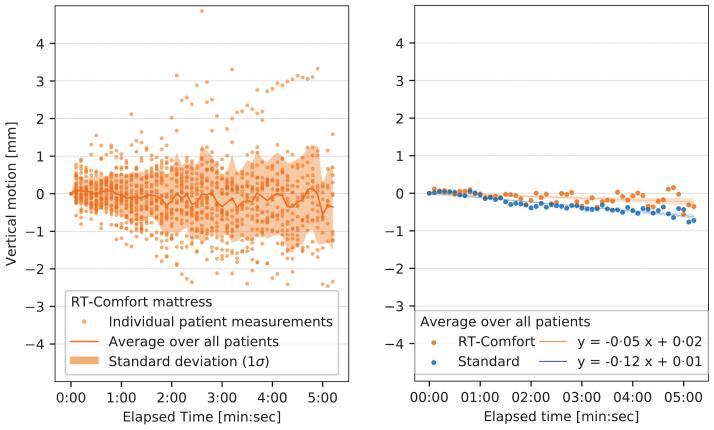
Vertical motion of patients during radiotherapy treatment. The left panel shows the motion analysis of the patients on the RTComfort mattress during radiotherapy treatment. The optical surface measurements were started directly after the setup correction based on the CBCT imaging. Every orange dot represents the median motion of one patient during a 6-s time bin, with n = 31 patients at the start. The orange line is the mean vertical motion over all patients, with the standard deviation indicated by the filled area around the mean. As some treatments are shorter than others, the number of patients declined over time. The graph shows only data for time points with >10 patients in the RTComfort group. The right panel shows the mean vertical motion over all patients in the RTComfort group (again, orange) and the historical cohort (n = 128) fitted by linear regression lines with 95 % confidence intervals. (For interpretation of the references to colour in this figure legend, the reader is referred to the web version of this article.)

Out of 45 patients included in the clinical study, 31 successful optical surface measurements could be collected, as for 9 patients the signal was not recorded (including the single patient treated on the standard matt) and for 4 patients the recording was not according to the protocol. One patient was excluded because of an implausible raise of more than 1 cm after positioning. From the historical cohort the optical surface data of 128 patients could be evaluated. As some treatments are shorter than others, the number of patients declined as the measurement time increased. The last time point with at least 10 patients in the clinical study was 5 min and 12 s. At the same time point, 25 patients were remaining in the reference cohort. The results of the surface monitoring of the clinical study are shown in Fig. 2A. Fig. 2B shows the mean vertical position fitted by linear regression lines with 95 % confidence intervals. Linear regression analysis showed negligible sagging coefficients of −0.05 mm/min on the RTComfort (orange) and −0.12 mm/min on the standard matt (blue). The difference of −0.07 mm/min between both (*interaction term*), was statistically significant with p < 0.0001, indicating that the RTComfort led to slightly less sagging.

#### Dosimetric evaluation

The 44 patients scanned on the RTComfort mattress were treated with 50 different treatment plans. Six patients had two treatment plans for multiple targets. The mean dose to the planning target volume (PTV) was 101.0 % (SD 0.4 %) of the prescribed dose, with a mean V95% coverage of 99.3 % (SD 0.7), and maximum skin dose of 82.7 % (SD 16.3 %) of the prescribed dose. When recalculated while accounting for the mattress, these values changed slightly to 100.1 % (SD 0.5 %), 99.0 % (SD 0.9 %) and 88.5 % (SD 16.4) respectively. Re-optimization with the RTComfort mattress fully recovered these values to 101.0 % (SD 0.3 %), 99.1 % (SD 0.8 %) and 80.8 % (SD 16.7 %).

## Discussion

In radiotherapy flat and hard treatment couches to allow reproducible patient positioning and accurate dose delivery. In palliative setting, a thin foam matt is often used to reduce discomfort. This study described the development and clinical evaluation of the RTComfort mattress (patent pending) to enhance comfort for patients with painful metastasis during radiotherapy preparation and treatment. The RTComfort mattress has a thickness of 6 cm and was compared against the standard thin foam matt (2 cm). In total, 98 % of patients preferred the RTComfort mattress over the regular matt and the median experienced pain intensity dropped by 2.8 (NRS, 0–10 pain scale) which is considered clinically significant [18]. The positioning stability of patients on the RTComfort mattress was slightly better compared to the standard matt, possibly due to the reduced experienced discomfort and pain [8]. The RTComfort mattress had a negligible impact on attenuation and skin dose, which could be recovered completely by accounting for the RTComfort mattress during treatment planning. From a more practical perspective, the RTComfort mattress was compatible with armrests, knee and feet support, and the mattress ticking was easy to clean. These results demonstrate that the RTComfort mattress is safe to use, and it can offer comfort and pain reduction in patients receiving palliative radiotherapy treatments. At present already more than 500 patients have been treated on the RTComfort mattress in our center. The mattress is fully developed and ready for production and commercialization. We expect the study to lead to a worldwide paradigm shift where all radiotherapy patients that experience pain will be treated on comfortable mattresses.

Although the urge for increase in comfort during palliative radiotherapy treatments is established in literature [8,9,12] to the best of our knowledge, there is little literature evaluating the patients’ comfort and experienced pain during radiotherapy [19]. In 2004, Pignon and colleagues scored the mean pain intensity according to the same Numerical Rating Scale (NRS) during the waiting time right before the radiotherapy treatment session. In a single-day cross-sectional study in 93 patients (of which 77 were treated with a curative intent) they found a mean pain intensity of 3.9 (SD 2.3, NRS) [20]. This is comparable but lower than the pain scored in the patients treated on the standard matt in our cohort, which was 4.8 (IQR: 2.0 to 6.1, NRS), however, it is surprisingly higher than the median pain score of 2.0 (IQR: 0.4 to 4.0, NRS) as scored by the patients with palliative intent on the RTComfort mattress.

Our findings may support investigation of the potential benefit for patients undergoing curative treatments as well, especially in longer-lasting online adaptive treatments [7]. Moreover, the observed (sub)millimeter patient positioning accuracy would allow for curative radiotherapy, or even stereotactic treatments, without additional correction for intra-fraction motion [21,22]. The patient positioning stability was reported within the first 5 min after treatment positioning and positioning verification. Based on the water barrel phantom and volunteer measurements that took 32 min, no decrease in stability is expected in a timeframe of up to 30 min.

In conclusion, this prospective clinical study demonstrated successful development, implementation and evaluation of a new RTComfort mattress for palliative radiotherapy treatments. The overwhelming majority of patients (44 out of 45) preferred the RTComfort mattress over the standard matt and the RTComfort mattress led to a clinically significant pain reduction, without affecting positioning stability or treatment accuracy. Therefore, the RTComfort mattress is safe to use, and it can offer comfort and pain reduction in patients receiving palliative radiotherapy treatments. In our institute the RTComfort mattress (patent pending) is now released for clinical use for all eligible patients treated with palliative intent. Moreover, our findings support further deployment of the RTComfort mattress, also beyond the palliative setting and radiotherapy. We expect that the study could lead to a broader adaptation of more comfortable mattresses for all patients that experience pain during radiotherapy delivery.

## CRediT authorship contribution statement

**Nienke Hoffmans-Holtzer:** Conceptualization, Formal analysis, Investigation, Methodology, Validation, Writing – original draft. **Britt Kunnen:** Project administration, Validation, Investigation, Writing – original draft. **Olijn Tims:** Data curation, Resources. **Ilse de Pree:** Data curation, Resources. **Cleo Slagter:** Data curation, Resources. **Manouk Olofsen-van Acht:** Conceptualization, Data curation, Resources, Writing – review & editing, Supervision. **Mischa Hoogeman:** Conceptualization, Resources, Writing – review & editing, Supervision. **Steven Petit:** Conceptualization, Methodology, Resources, Writing – review & editing, Supervision.

## Declaration of competing interest

The authors declare the following financial interests/personal relationships which may be considered as potential competing interests: The department of Radiotherapy of the Erasmus MC Cancer Institute has research collaborations with Royal Health Foams (Den Haag, The Netherlands), Elekta (Elekta AB, Stockholm, Sweden), Accuray Inc. (Sunnyvale, CA, USA), Varian (Siemens Healthineers company, Palo Alto, CA, USA), RaySearch Laboratories (Stockholm, Sweden), and Vision RT (London, UK).

The authors gratefully acknowledge Chris Nederhorst from Royal Health Foams for his experience on, and contributions to the development of the RTComfort mattress. Royal Health Foams provided prototypes and mattresses in kind.

Nienke Hoffmans-Holtzer, Olijn Tims and Steven Petit, are listed as inventors on the patent application.
