# Supplementary material for: Increasing patient comfort in palliative radiotherapy with a newly developed mattress: a nonrandomized clinical trial
Source: Clin Transl Radiat Oncol. 2025 Jul 16;54:101017. doi: 10.1016/j.ctro.2025.101017 (PMC12296526; doi:10.1016/j.ctro.2025.101017)
Supplement: Supplementary Data 1 [file mmc1.pdf]

Supplementary Materials

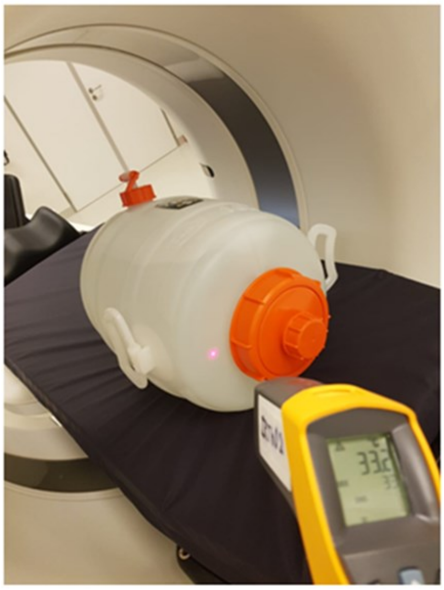

Figure S1: *Setup of the pre-heated water phantom CT-measurement for testing of the standard matt and prototype mattresses.*

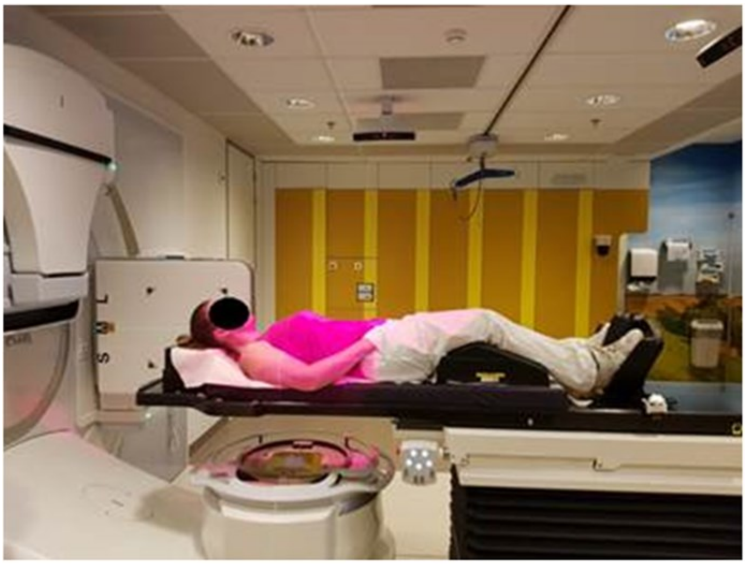

Figure S2: *Setup of the volunteers and patient measurements at the radiotherapy treatment machine, with patients positioned on the mattress on the treatment table and SGRT cameras on the ceiling.*

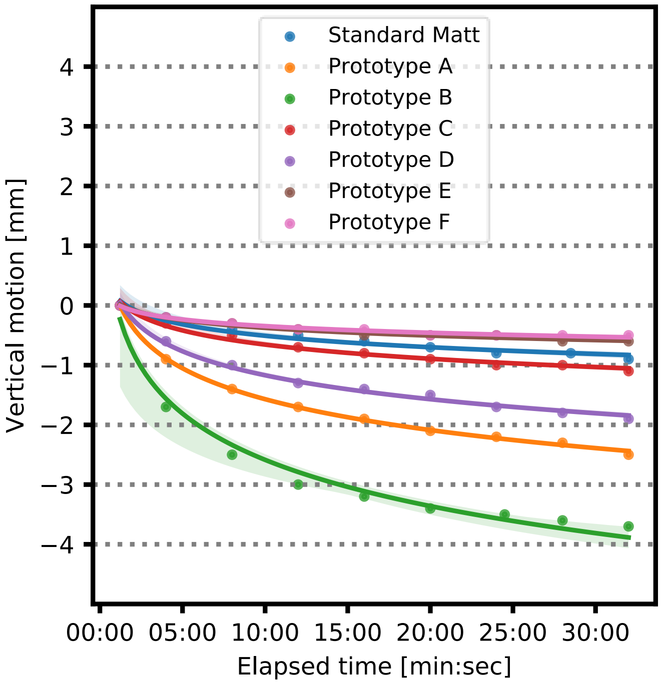

Figure S3: *Results of the standard matt and prototype mattresses CT-measurements using a pre-heated water phantom (see Figure S1). Dots are fitted by loglinear curves with 95% confidence intervals.*

		PTV				Skin		
	**MU**	**95% cov [%]**	**Min [Gy]**	**Mean [Gy]**	**Max [Gy]**	**Mean [Gy]**	**Max [Gy]**	**Attenuation**
**3D-Conformal (single PA beam)**								
Planned w/o RTComfort	735.1	95.1	6.6	8.6	10.1	2.8	8.8	
Recalculated with RTComfort	735.1	91.2	6.5	8.5	10.0	4.1	10.1	1.4%
Rescaled with RTComfort	746.2	95.0	6.6	8.6	10.1	4.1	10.3	
**VMAT (rotational delivery)**								
Optimized w/o RTComfort	1108.5	98.4	7.2	8.1	8.6	2.1	4.5	
Recalculated with RTComfort	1108.5	97.2	7.0	8.0	8.5	2.8	8.3	0.9%
Re-optimized with RTComfort	1149.9	98.0	7.2	8.1	8.7	2.9	5.1	

Tabel S1: *Results of dosimetric evaluation of the pre-heated water barrel phantom with and without accounting for the RTComfort mattress. Attenuation of the RTComfort mattress is calculated as mean PTV dose without RTComfort divided by the mean PTV dose with accounting for the RTComfort mattress.*

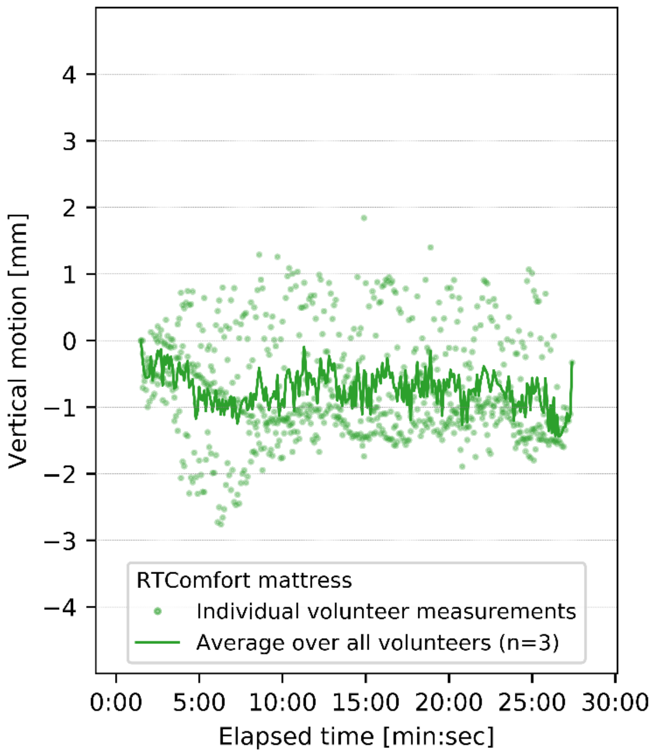

Figure S4: *Vertical motion analysis of three volunteers on the RTComfort mattress. The AlignRT® measurements were started two minutes after the volunteer-positioning, mimicking the time used for CBCT imaging in clinical patients. Every green dot represents the median motion of one patient during a 6-sec time bin. The green line is the mean vertical motion over all three volunteers.*
